# A versatile transposon-based technology to generate loss- and gain-of-function phenotypes in the mouse liver

**DOI:** 10.1186/s12915-022-01262-x

**Published:** 2022-04-01

**Authors:** Anna Georgina Kopasz, Dávid Zsolt Pusztai, Réka Karkas, Liza Hudoba, Khaldoon Sadiq Ahmed Abdullah, Gergely Imre, Gabriella Pankotai-Bodó, Ede Migh, Andrea Nagy, András Kriston, Péter Germán, Andrea Bakné Drubi, Anna Molnár, Ildikó Fekete, Virág Éva Dani, Imre Ocsovszki, László Géza Puskás, Péter Horváth, Farkas Sükösd, Lajos Mátés

**Affiliations:** 1 Institute of Genetics, Biological Research Centre, Szeged, Hungary; 2 Doctoral School of Biology, University of Szeged, Szeged, Hungary; 3 Doctoral School of Multidisciplinary Medical Sciences, University of Szeged, Szeged, Hungary; 4 Institute of Pathology, University of Szeged, Szeged, Hungary; 5 Synthetic and Systems Biology Unit, Institute of Biochemistry, Biological Research Centre, Szeged, Hungary; 6 Department of Biochemistry, University of Szeged, Szeged, Hungary; 7 Institute for Molecular Medicine Finland (FIMM), University of Helsinki, Helsinki, Finland

**Keywords:** *Fah* KO mouse, *Sleeping Beauty*, Somatic transgenesis, In vivo gene silencing, Tumor model

## Abstract

**Background:**

Understanding the contribution of gene function in distinct organ systems to the pathogenesis of human diseases in biomedical research requires modifying gene expression through the generation of gain- and loss-of-function phenotypes in model organisms, for instance, the mouse. However, methods to modify both germline and somatic genomes have important limitations that prevent easy, strong, and stable expression of transgenes. For instance, while the liver is remarkably easy to target, nucleic acids introduced to modify the genome of hepatocytes are rapidly lost, or the transgene expression they mediate becomes inhibited due to the action of effector pathways for the elimination of exogenous DNA. Novel methods are required to overcome these challenges, and here we develop a somatic gene delivery technology enabling long-lasting high-level transgene expression in the entire hepatocyte population of mice.

**Results:**

We exploit the fumarylacetoacetate hydrolase (Fah) gene correction-induced regeneration in Fah-deficient livers, to demonstrate that such approach stabilizes luciferase expression more than 5000-fold above the level detected in WT animals, following plasmid DNA introduction complemented by transposon-mediated chromosomal gene transfer. Building on this advancement, we created a versatile technology platform for performing gene function analysis in vivo in the mouse liver. Our technology allows the tag-free expression of proteins of interest and silencing of any arbitrary gene in the mouse genome. This was achieved by applying the HADHA/B endogenous bidirectional promoter capable of driving well-balanced bidirectional expression and by optimizing in vivo intronic artificial microRNA-based gene silencing. We demonstrated the particular usefulness of the technology in cancer research by creating a p53-silenced and hRas G12V-overexpressing tumor model.

**Conclusions:**

We developed a versatile technology platform for in vivo somatic genome editing in the mouse liver, which meets multiple requirements for long-lasting high-level transgene expression. We believe that this technology will contribute to the development of a more accurate new generation of tools for gene function analysis in mice.

**Supplementary Information:**

The online version contains supplementary material available at 10.1186/s12915-022-01262-x.

## Background

Genetic manipulations to modify gene expression in a cell or an organism have been applied in numerous fields including research, medicine, industrial biotechnology, and agriculture. In research, they are used to study gene function through the generation of loss-of-function and gain-of-function phenotypes, to characterize gene expression patterns through the introduction of reporter genes, and to visualize intracellular trafficking of macromolecules through mRNA and protein tagging. The laboratory mouse is currently the predominant mammalian species in biomedical research, most commonly used as an experimental model system for investigating the pathogenesis of human diseases and for developing new therapies. However, genetic manipulations in the mouse germline are generally laborious and time consuming and frequently result in unstable transgene expression or unreliable spatiotemporal expression pattern [1, 2]. Alternatively, if genetic modifications target the soma of mice, the workflows are faster. However, these workflows are becoming dominated by viral gene delivery, and in turn, they are hampered by viral cargo limitations, and despite natural viral infectivity, they often result in unstable gene expression due to host immune response against viral proteins [3, 4]. To replace viruses, lipid nanoparticles are used extensively as synthetic non-viral delivery vehicles. They are less prone to trigger host immune response than viral vectors due to the absence of immunogenic viral proteins and do not exhibit strict cargo limitations. However, lipid nanoparticle-based somatic gene delivery is generally not as effective as the viral one. Different organs can be targeted with varying degrees of efficiency, but in the case of the liver, the procedure is particularly effective [5]. The liver can also be efficiently targeted with naked plasmid DNA using a simple in vivo transfection procedure called hydrodynamic injection [6].

However, transgene expression rapidly declines in the liver following plasmid DNA delivery [7]. To date, a number of receptors have been reported to recognize cytosolic exogenous DNA, such as Toll-like receptor 9 (TLR9), cyclic GMP-AMP synthase (cGAS), and absent in melanoma 2 (AIM2) inflammasomes [8, 9]. These receptors trigger effector pathways that contribute to the elimination and transcriptional repression of plasmid DNA. To improve the outcome of plasmid DNA delivery, the system can be supplemented with non-viral transposon-based chromosomal gene transfer. Indeed, together with *Sleeping Beauty* (SB) transposon-mediated chromosomal transfer of the transgene, the long-term parameters of gene expression slightly improve, but a few weeks after injection, the decrease in transgene expression remains enormous [10]. This suggests that the effect of cytosolic DNA sensors can only be partially avoided, even if the technology is complemented by chromosomal gene transfer. Problems persist and transgene expression is not stably maintained, at least not to a sufficient extent.

Our aim was to change this situation by improving the current biotechnology toolkit and harnessing the SB transposon together with an efficient somatic transgenesis system, taking the efficiency, versatility, and stability of liver-specific gene delivery in mice to the next level. We planned to create a technology that would simultaneously allow the expression of native or mutant isoforms of proteins and efficient silencing of any arbitrary target gene in the mouse genome, in a stable manner and a high number of cells. We took the advantage of hydrodynamic injection for efficient in vivo transfection of hepatocytes and hyperactive SB [11] transposon-mediated chromosomal gene transfer for stable transgene delivery. For achieving high affected cell number and high stability of transgene expression, we harnessed the known selection pressure exerted in fumarylacetoacetate hydrolase (*Fah*) KO livers for Fah-expressing hepatocytes [12]. Finally, for more versatile gene expression modifications, we applied a new bidirectional promoter that had not previously been part of the biotechnology toolkit and optimized intron-derived microRNA (miR)-based gene silencing in vivo in the mouse liver.

The technology platform reported here is well suited to study gene function in hepatocytes via the generation of gain-of-function and loss-of-function phenotypes as it allows the exchange of virtually the entire hepatocyte population for transgenic cells. Here we demonstrate its particular usefulness in cancer research. A current high priority in cancer research is to functionally validate candidate genetic alterations that are relevant for cancer progression as it is no longer possible to clearly identify them using bioinformatics methods based on mutation frequency analysis alone, due to potential candidates being mutated at lower frequencies in cancer samples. Thus, there is a growing demand for in vivo experimental systems where the cancer driver role of mutations could be confirmed.

## Results

### Simple and highly efficient gene delivery system for directed gene expression modifications in the mouse liver

**Fig. 1:**
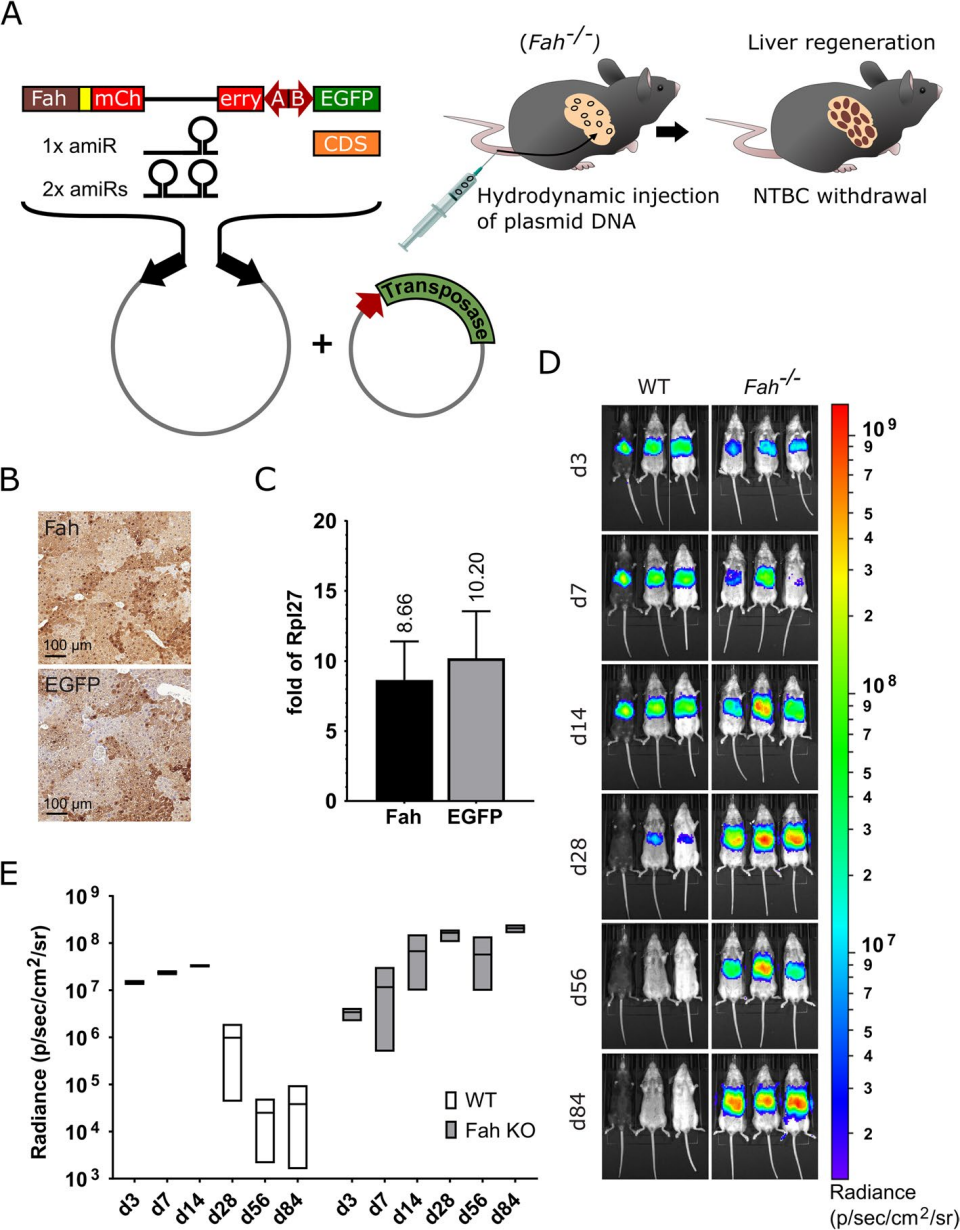
In vivo transposon-based gene delivery into the liver of *Fah*^−/−^ and WT mice. **a** Schematic representation of the *Sleeping Beauty* (SB) transposon-based cloning platform and animal treatments. Black arrows, SB transposon inverted terminal repeats; red arrows, promoters. **b** Fah and EGFP immunostainings of liver sections from *Fah*^−/−^ mice 3 months after NTBC withdrawal. **c** Monitoring the amount of transcripts A and B following in vivo gene delivery. Liver RNA samples were collected from *Fah*^−/−^ mice at 3 months post-treatment. Samples were tested using Fah- and EGFP-specific RT-qPCR assays. Results were normalized to measurements of the ribosomal protein L27 (*Rpl27*) transcript as input control and data were presented as the mean ± standard deviation (SD) (*n* = 3) (see Additional file 2 for individual data values and statistics). **d** Live bioluminescence imaging of *Fah*^−/−^ and WT mice following in vivo gene delivery. Bioluminescence signals were obtained using an IVIS Lumina III imaging system at 3, 7, 14, 28, 56, and 84 days post-treatment. **e** Kinetics of bioluminescence changes during the first 3 months after gene delivery. For each experimental animal, the average radiance (photons/second/cm^2^/steradian (sr) [p/s/cm^2^/sr]) of circular regions of the same size covering the liver area was used for plotting. The numerical values were presented as box diagram from lowest to highest values with line at mean (*n* = 3) (see Additional file 2 for individual data values and statistics)

Keeping in mind the general simplicity of the procedure, we based our system on the hydrodynamic injection of plasmid DNA constructs. Hydrodynamic plasmid delivery primarily targets hepatocytes by the enhancement of their membrane permeability [6, 13]. However, following in vivo transfection, chromosomal integration is also required for long-term stable transgene expression. For very efficient SB transposon-based chromosomal transgene delivery, we used a hyperactive transposase helper SB100 [11] and a more active transposon, the so-called T2 Inverted Terminal Repeat (ITR) structure variant [14]. In those hepatocytes where hydrodynamic transfection is successful, the hyperactive transposase helper enzyme is likely to catalyze the “cut and paste” transposition reaction presumably leading to an integration into the host chromosomes [15]. However, literature data suggest that the negative effect of exogenous DNA sensors on transgene expression cannot be efficiently avoided, even if the technology is complemented by chromosomal gene transfer [10]. We hypothesized that a high level of sustained transgene expression could be achieved by exploiting the well-known high regenerative potential of adult mouse hepatocytes. Following an extended hepatectomy, which involves removing nearly 90% of liver tissue, the mouse liver can regenerate within a short time regaining its normal size [16]. Such a high regenerative potential can be harnessed to replace virtually all *Fah*^−/−^ hepatocytes for *Fah*^+/+^ ones in a *Fah*-deficient liver by multi-nodular repopulation due to the selective growth advantage of wild-type (WT) cells [12]. Therefore, we supplemented our transposon construct with a *Fah* coding sequence (CDS) and used a *Fah* mutant mouse strain (C57BL/6N-*Fah*^*tm1(NCOM)Mfgc/Biat*^) for the hydrodynamic injections (Fig. 1a).

For directed gene expression modifications, we constructed an SB transposon-based cloning platform (Fig. 1a) built on the co-expression of two linked transcripts allowing transgene expression to be bound to the expression of the *Fah* selection marker. Transcript A codes for the selection and visualization marker proteins connected by a “self-cleaving” T2A peptide for bicistronic expression [17], whereas transcript B encodes the protein to be tested without the need for tagging. To this end, we complemented the current biotechnology toolbox with the human *HADHA/B* promoter capable of driving well-balanced bidirectional expression. The compact (390 bp) *HADHA/B* bidirectional promoter drives the expression of the human hydroxyacyl-CoA dehydrogenase trifunctional multienzyme complex alpha (HADHA) and beta (HADHB) subunits [18].

To test the functionality of the design, an EGFP-encoding transcript B driven by the *HADHB* side of the bidirectional promoter was created (Fig. 1a). First, this EGFP-expressing transposon and the SB100 transposase helper plasmid were co-delivered hydrodynamically into the liver of *Fah*^−/−^ mice, then the curative drug nitisinone (NTBC**)** [12] that had been given continuously up to that point was withdrawn. Immunohistochemical (IHC) investigations (Fig. 1b) demonstrated that due to intensive multi-nodular repopulation after 3 months, virtually all hepatocytes were Fah- and EGFP-positive in the treated livers. Next, using a quantitative reverse transcription PCR (RT-qPCR) method, we identified the amount of transcripts A and B produced by the *HADHA* and *HADHB* sides of the promoter, respectively. The amounts of mCherry-T2A-Fah and EGFP mRNAs were normalized to that of the ribosomal protein L27 (*Rpl27*). Our RT-qPCR data demonstrate that the transgene had an order of magnitude stronger expression than the ribosomal protein Rpl27 in the liver (Fig. 1c), which is fully consistent with a strong but physiological gene expression level. It should be noted that the expression levels of transcripts A and B were virtually identical (1/1.18).

To better explore the impact of multi-nodular repopulation on long-term transgene expression in *Fah*-deficient livers, the same construct, in which transcript B was coding for the firefly luciferase (Luc) marker protein (Fig. 1a), was injected into both wild-type (WT) and *Fah* KO mice in the presence of the SB100 transposase helper. Transgene expression was monitored over time by detecting the bioluminescence in living animals using the IVIS Lumina III imaging system (PerkinElmer). After a slightly higher Luc expression detected in WT animals during the first week after hydrodynamic injection, this transgene expression trend was reversed from day 28 onwards. In 3 months, by day 84, the average bioluminescence intensity in WT animals decreased to 387-fold of the initial level, while in *Fah* KO animals it reached 61-fold of the initial level (Fig. 1d, e).

### Design and in vivo application of amiR elements

**Fig. 2:**
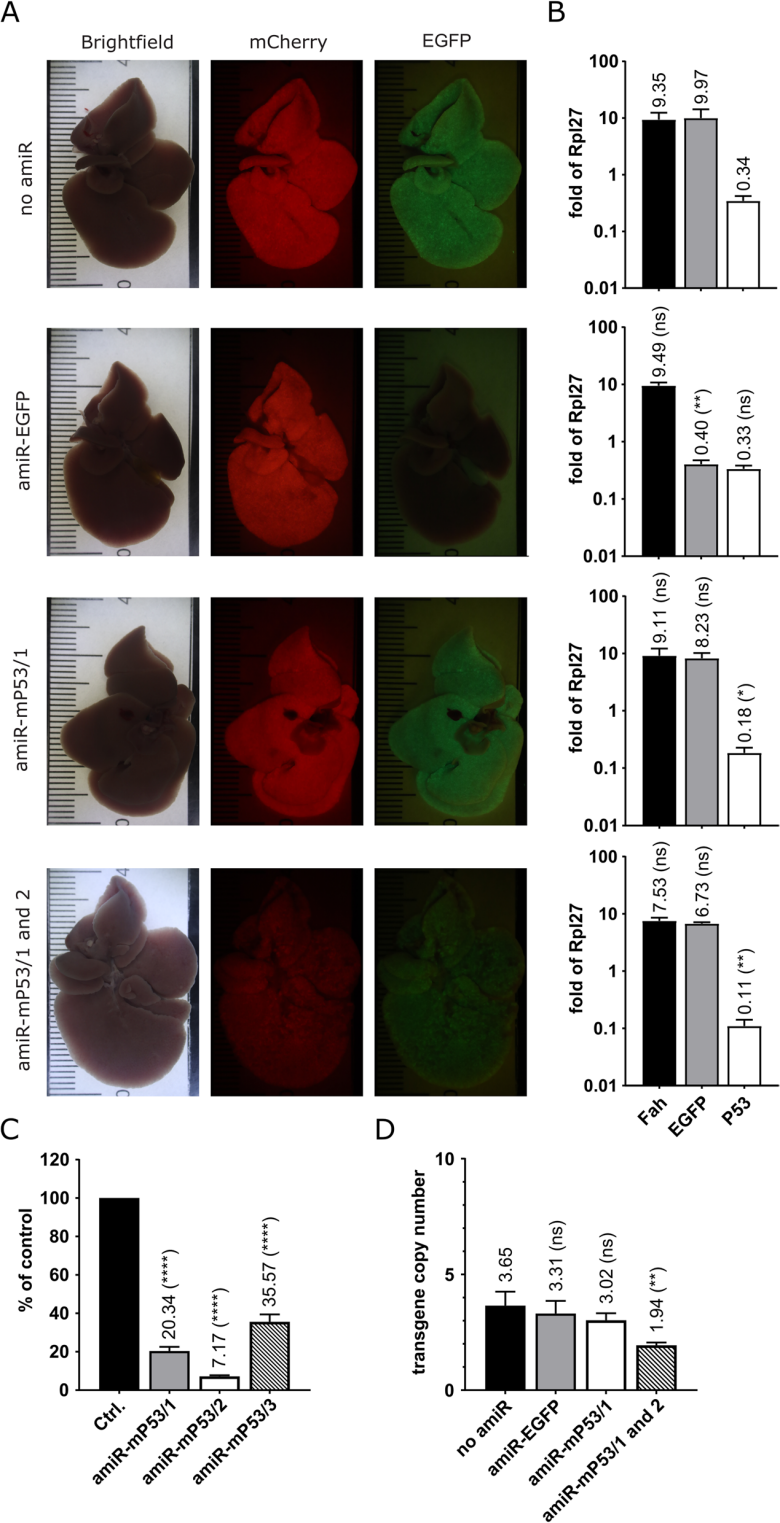
In vivo amiR-based gene silencing in the mouse liver. **a** Brightfield and fluorescence stereomicroscopic images of the liver of *Fah*^−/−^ mice 5 months after the intrahepatic delivery of an amiR-free control and different amiR-expressing transposon vectors. **b** Monitoring the amount of the endogenous p53 mRNA and artificial transcripts A and B in the liver of *Fah*^−/−^ mice 5 months after intrahepatic delivery of an amiR-free control and different amiR-expressing transposon vectors. Liver RNA samples were collected from *Fah*^−/−^ mice at 5 months post-treatment. Samples were tested using Fah-, EGFP-, and p53 mRNA-specific RT-qPCR assays. Results were normalized to measurements of the ribosomal protein L27 (*Rpl27*) transcript as input control and data were presented as the mean ± SD (*n* = 3) (see Additional file 2 for individual data values and statistics). **c** Monitoring of endogenous p53 mRNA levels in NIH3T3 cells after stable transposon-based delivery of different amiR elements designed to silence *Tp53* expression. RNA samples were collected from cultured cells after G418 selection and tested using a p53 mRNA-specific RT-qPCR assay. Results were normalized to measurements of the ribosomal protein L27 (*Rpl27*) transcript as input control. Data were presented as the mean ± SD as relative values compared to the value generated using an amiR-free control vector (*n* = 3) (see Additional file 2 for individual data values and statistics). **d** Copy numbers of the transgenes in the liver of *Fah*^−/−^ mice following intrahepatic delivery of different transposon vectors. Liver DNA samples were collected from *Fah*^−/−^ mice at 5 months post-treatment. Samples were tested using a *Fah* transgene-specific qPCR assay. Results were normalized to measurements of the olfactory receptor 16 (*Olfr16*) gene as an input control, and values were presented relative to one diploid genome (*n* = 3) (see Additional file 2 for individual data values and statistics)

Our SB transposon-based cloning platform also provides an opportunity for simultaneous silencing of endogenous genes by the expression of artificial microRNA (amiR) elements. In order to avoid interference with the translation of marker proteins, amiR structures were expressed from an intron inserted into transcript A. To accommodate intronic amiR structures, the modified 943-bp-long first intron of the human eukaryotic translation elongation factor 1 alpha 1 (*EEF1A1*) gene was incorporated in the mCherry CDS (Fig. 1a). To verify the in vivo applicability of intronic amiR elements in our platform, we first incorporated an amiR element silencing EGFP (amiR-EGFP) into the intron of the mCherry CDS. In this arrangement, amiR-EGFP, which is processed from transcript A, silences EGFP encoded by transcript B. The guide sequence of amiR-EGFP targeting the EGFP CDS was designed by Beisel et al. [19]. For the creation of the amiR-EGFP, “miR-E,” an optimized human miR-30a-based miR backbone, was applied [20], and the EGFP guide sequence was inserted into the miR-E backbone structure (Additional file 1: Fig. S1). This amiR-EGFP- and EGFP-expressing transposon and the SB100 transposase helper plasmid were co-delivered into the liver of *Fah*^−/−^ mice, then NTBC was withdrawn. After 5 months, following complete multi-nodular repopulation, the animals were sacrificed. Macrovisualization of mCherry and EGFP autofluorescence in the liver showed that silencing of EGFP expression was very effective compared to control animals without amiR structures (Fig. 2a). The results of our EGFP Western blot assays also confirmed effective knockdown at the protein level (Additional file 1: Fig. S2). Next, RT-qPCR measurements were used to determine the amount of A and B transcripts. According to these measurements, transcript B encoding EGFP was silenced to 4% residual gene expression in livers expressing amiR-EGFP elements (Fig. 2b).

After successful in vivo silencing of EGFP, we selected an endogenous target gene, *Tp53*, for amiR-mediated silencing. For amiR-mP53 structures — similarly to amiR-EGFP — guide sequences targeting endogenous p53 mRNA were inserted into the “miR-E” backbone (Additional file 1: Fig. S1). The amiR-mP53 guide sequences were designed following the guide design rules of Dow et al. [21] summarized in Additional file 1: Fig. S1.

To test the efficacy of amiR-mP53 elements designed to silence mouse *Tp53*, we performed studies in a mammalian tissue culture system. To this end, we inserted the first intron of the EEF1A1 gene, modified to accommodate amiR elements, into the sequence encoding the Neomycin selection marker protein and then incorporated the three amiR-mP53 elements one by one into this intron. The resulting Neomycin expression units were then inserted between the SB transposon ITRs (Additional file 1: Fig. S3). Next, the transposon series carrying the different amiR-mP53 elements and an amiR-free control transposon were co-transfected with the SB100 transposase helper plasmid into NIH3T3 cells. After G418 selection, RT-qPCR measurements were performed to determine the level of endogenous p53 mRNA in the transfected cells. Our measurements showed that all three amiR-mP53 elements were sufficiently effective. However, amiR-mP53/1 and amiR-mP53/2 elements performed better, producing 20.34 and 7.17% remaining gene expression, respectively, and were used for further in vivo studies (Fig. 2c). Subsequently, amiR-mP53/1 alone or amiR-mP53/1 and amiR-mP53/2 elements together were incorporated into the intron of the mCherry CDS in our cloning platform designed for in vivo studies (Fig. 1a). These amiR-mP53-expressing transposons and the SB100 transposase helper plasmid were co-delivered hydrodynamically into the liver of *Fah*^−/−^ mice, then NTBC was withdrawn. After 5 months, following complete multi-nodular repopulation, the animals were sacrificed. Macrovisualization of the mCherry and EGFP autofluorescence showed that in these organs the expression of the mCherry and EGFP marker proteins was balanced and comparable to the control (Fig. 2a). Our RT-qPCR measurements confirmed that transcript A and B levels were not significantly altered compared to the control (Fig. 2b). However, we found 53% remaining *Tp53* expression in organs expressing amiR-mP53/1, while organs co-expressing amiR-mP53/1 and amiR-mP53/2 showed 32% remaining *Tp53* expression (Fig. 2b). This is in line with expectations, as hepatocytes make up about half of liver tissue [22], the other cell types are not affected by *Tp53* silencing. In contrast, EGFP is expressed exclusively in hepatocytes and therefore all EGFP-expressing cells are affected by gene silencing.

With successful gene silencing, the liver regeneration of animals expressing amiR was similar to that of controls without amiR, and no signs of non-specific toxicity associated with amiR expression were observed. The slight decrease in transgene copy number observed in livers expressing one or two amiR-mP53 elements (Fig. 2d) may explain the slight, non-significant decrease in transcript A and B levels in these organs as compared to controls (Fig. 2b).

### Hepatocellular carcinoma modeling using predefined combinations of drivers

**Fig. 3:**
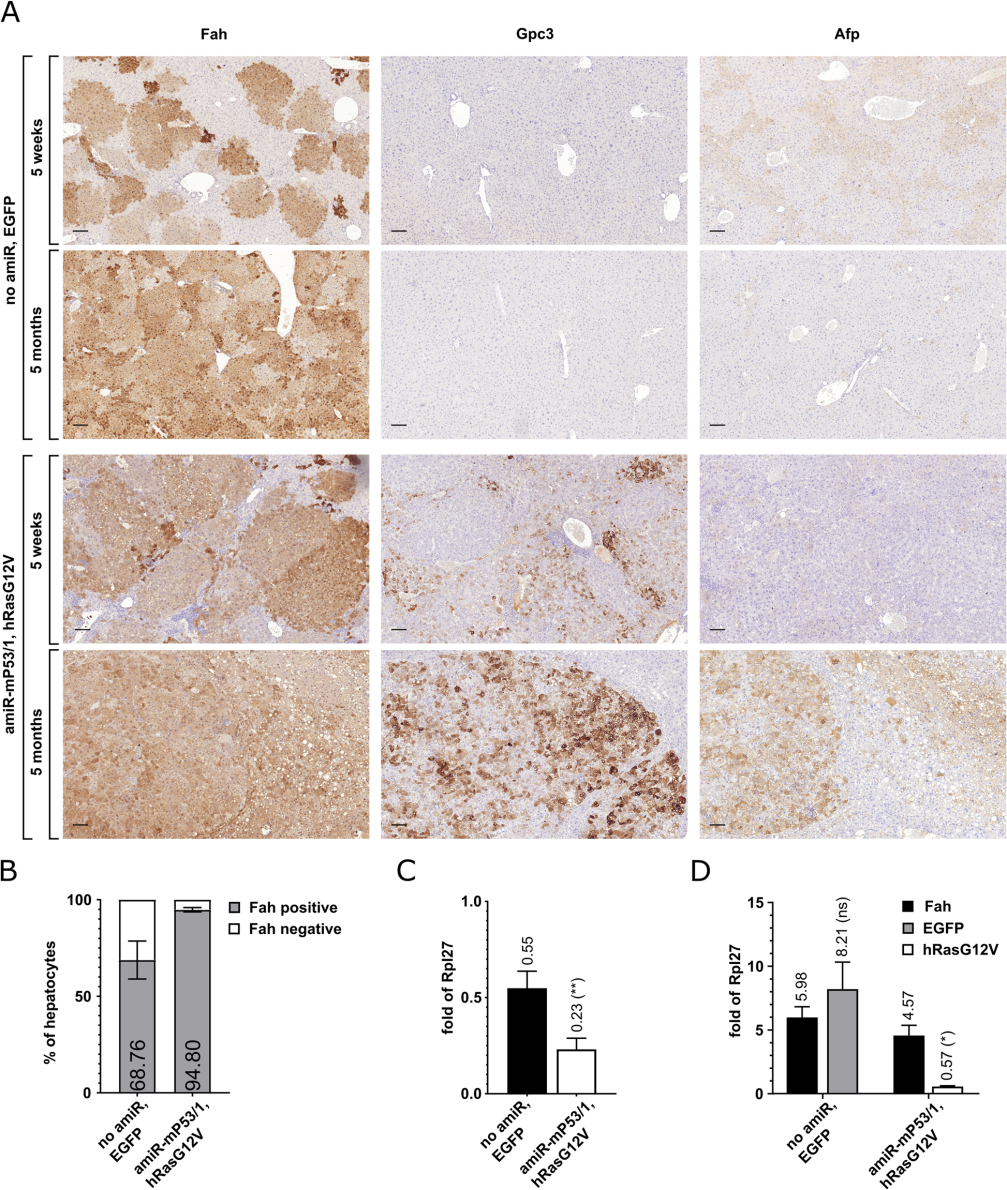
Induction of HCC using a predefined combination of drivers. **a** Immunohistochemical analysis of the Fah selection marker, Gpc3, and Afp HCC markers in liver sections from *Fah*^−/−^ mice treated with either control (no amiR, EGFP) or driver (amiR-mP53/1, hRas^G12V^) transposon constructs at 5 weeks and 5 months post-treatment. For the analysis of tumors emerging 5 months after treatment with the driver construct, a vector mixture containing 1% driver transposon vector and 99% transposon vector expressing only the Fah selection marker protein was used. Scale bars, 100 μm. **b** Determination of the percentage of Fah-positive hepatocytes 5 weeks after treatment by machine learning-based measurement. Data were presented as the mean ± SD (*n* = 3) (see Additional file 2 for individual data values and statistics). **c** Monitoring of endogenous p53 mRNA levels in the liver of *Fah*^−/−^ mice treated with driver and control transposon constructs. Liver RNA samples were collected from *Fah*^−/−^ mice at 5 weeks post-treatment and tested using a p53 mRNA-specific RT-qPCR assay. Results were normalized to measurements of the ribosomal protein L27 (*Rpl27*) transcript as input control and data were presented as the mean ± SD (*n* = 3) (see Additional file 2 for individual data values and statistics). **d** Monitoring the amount of transcripts A and B in the liver of *Fah*^−/−^ mice treated with driver and control transposon constructs. Liver RNA samples were collected from *Fah*^−/−^ mice at 5 weeks post-treatment and tested using Fah-, EGFP-, and hRas^G12V^-specific RT-qPCR assays. Results were normalized to measurements of the ribosomal protein L27 (*Rpl27*) transcript as input control and data were presented as the mean ± SD (*n* = 3) (see Additional file 2 for individual data values and statistics)

To demonstrate the utility of our technology in cancer research and the ability to co-express mutant or native proteins with amiR elements, in our cloning platform, we created an SB transposon armed to silence the endogenous *Tp53* and overexpress an oncogenic hRas variant. To achieve this, amiR-mP53/1 was introduced into the intron of the mCherry CDS in transcript A and hRas^G12V^ — a constitutively active form of hRas [23] — was built into transcript B (Fig. 1a). Next, the amiR-mP53/1- and hRas^G12V^-expressing driver and the amiR-free EGFP-expressing control transposon constructs together with the SB100 transposase helper plasmid were co-delivered hydrodynamically into the liver of *Fah*^−/−^ mice, then NTBC was withdrawn. After 5 weeks, during multi-nodular repopulation, the animals were sacrificed. Immunohistochemical investigations showed that the size of Fah-positive hepatocyte colonies repopulating the liver was larger in livers treated with the driver construct as compared to controls (Fig. 3a). This is consistent with the significantly higher proportion of Fah-positive hepatocytes (94.8% vs. 68.76%) in the driver construct-treated animals as determined by machine learning technology (Fig. 3b). This clearly shows that the division rate of the transformed hepatocytes is markedly higher than that of the regenerating cells in control mice during normal multi-nodular repopulation.

The examination time point at 5 weeks after hydrodynamic injection and NTBC withdrawal represents incomplete multi-nodular repopulation. At this time point, we again monitored the extent of *Tp53* silencing and the levels of transcripts A and B in both driver and control construct-treated animals. Our RT-qPCR measurements revealed 42% remaining *Tp53* expression in organs treated with the driver construct (Fig. 3c). The levels of transcripts A and B in the livers of animals treated with the control construct were very similar to each other (Fig. 3d), comparable to the findings in the later stages following complete multi-nodular repopulation (Figs. 1 c and 2b), whereas a significant decrease in the expression of transcript B (hRas^G12V^) was observed in organs bearing the driver construct (Fig. 3d).

Experimental animals treated with the driver construct cannot be housed for substantially longer than 5 weeks due to the presence of a large number of transformed hepatocyte clones in their livers. Yet, to demonstrate the appearance of pathological signatures characteristic of tumors of malignant pathological grade using this high-penetrance driver combination, we mixed the transforming construct (1%) with a high amount of a transposon construct expressing the Fah selection marker protein alone (99%). Then, this construct mixture together with the SB100 transposase helper plasmid was co-delivered hydrodynamically into the liver of *Fah*^−/−^ mice. Treated mice sacrificed at 5 months after NTBC withdrawal exhibited a high tumor burden (Additional file 1: Fig. S4). On histological images of their livers, tumor tissue showed high intrinsic fat accumulation typical for hepatocellular carcinoma (HCC) [24, 25] and frequent neoplastic tissue invasion and atypical mitoses were observed (Additional file 1: Fig. S4).

To better characterize the induced tumorigenic processes, immunohistochemical investigations were performed on liver samples from control and driver construct-treated animals. An early time point, 5 weeks post-treatment, and a late time point, 5 months post-treatment, were chosen (Fig. 3a). Samples from animals treated with the 1% transforming construct mixture were used to investigate the long-term effect of the driver construct at 5 months post-treatment. All liver samples from control and driver construct-treated animals were analyzed by immunohistochemical staining for alpha-fetoprotein (Afp), the most commonly used marker of HCC [26], and glypican-3 (Gpc3), one of its early markers [27, 28] (Fig. 3a). Liver samples from mice treated with the control construct did not show positivity for the Gpc3 marker at any of the stages tested (Fig. 3a). In contrast, weak positivity for the Afp marker was observed mainly in early-stage control samples at 5 weeks post-treatment (Fig. 3a). This weak Afp signal is presumably produced in remaining tyrosinemic cells [29, 30]. Interestingly, in the driver construct-treated samples, the majority of transformed hepatocyte colonies already showed Gpc3 positivity at the earlier time point of 5 weeks post-treatment, while Afp positivity was not observed at this stage. Mature tumors, 5 months after treatment, typically showed the presence of both markers tested (Fig. 3a).

## Discussion

The manipulation of the somatic genome in mice is hampered by a number of factors that are essentially the same as those that make it difficult to manipulate the human somatic genome for gene therapy. Transcriptional repression observed with plasmid DNA vectors in the liver is caused by the formation of repressive heterochromatin on the plasmid DNA, a process initiated with the activation of cytoplasmic exogenous DNA sensors [8, 9]. Some studies suggest that heterochromatin formation occurs via CpG methylation [31, 32], whereas others propose a CpG-independent pathway [33]. As a consequence, transgene expression upon plasmid introduction, even if complemented by SB transposon-based chromosomal gene transfer rapidly declines in the liver [7, 10, 34]. Bell and co-workers demonstrated that even when using SB transposition, several weeks after injection, the transgene expression stabilizes at ∼1% of the level at 24 h [10]. According to the results of our own comparative experiment presented here, Luc expression stabilizes at 0.26% of the level measured at day 3 in WT animals (Fig. 1e).

In comparison to the standard hydrodynamic injection method into WT animals, we can state that by exploiting the multi-nodular repopulation in *Fah*-deficient livers, a truly long-lasting and physiological level of gene expression can be achieved, virtually in the entire hepatocyte population of the experimental mice (Fig. 1b, d, e). In *Fah*-deficient animals, Luc expression showed increasing intensity over the 3-month monitoring period, up to 61-fold above the initial level (Fig. 1d, e). This net increase arises from several contributing factors, some acting in opposite directions. The initial activation of DNA sensors presumably cannot be avoided even with the technology described here. Therefore, the main negatively contributing factor is probably transgene heterochromatinization, which almost completely abolishes transgene expression in WT animals. We speculate that using our technology, the transposed transgenes are unlikely to undergo the degree of heterochromatinization required for silencing, probably due to positive selection pressure on Fah marker expression and to cell divisions during liver regeneration. The transient decrease in transgene expression in *Fah* KO animals at day 56 is presumably due to the fact that these downregulation processes are also at work in them, peaking at around day 56 but eventually ceasing (Fig. 1d, e). Another negative contributor is the disappearance of plasmid-derived transgene expression, which is due, among other things, to the loss of plasmids during cell divisions. Presumably, this phenomenon accounts for the lower initial (d3, d7) bioluminescence values measured in *Fah* KO animals (Fig. 1d, e). The main factor positively affecting cumulative transgene expression is the division of transgene-bearing cells. The 61-fold increase in transgene expression could be directly attributed to a similar increase in the number of hepatocytes, which corresponds to roughly 5 cell divisions, if negatively acting factors are excluded. However, as these are also present, more than 5 cell divisions are likely to occur during complete multi-nodular repopulation [12].

The SB transposon system has been used before for *Fah* correction [35]. Some laboratories have also taken significant steps forward to create a versatile gene expression modification system such as the one we present here [36–40]. The first study, and the one most similar to ours, was published by Wangensteen et al. [36]. The other similar studies typically use this vector system or its variants. The technological approach developed by Wangensteen et al. differs in some fundamental points from the one we describe here. The authors of the study used an early variant of amiR design [41] for endogenous gene silencing, which was later improved by others towards the mirE type structure [20]. Notably, this transcriptional unit expressing the early amiR variant was present on a separate transposon vector from the transposon vector expressing the Fah selection marker and the NRas^G12V^ oncogene. Given that the toxicity of amiR elements may be significant [42, 43], target gene silencing was likely to affect only a subset of Fah-corrected NRas^G12V^ expressing cells in regenerating liver tissue. Although this potentially low double feature positive cell count works with penetrant tumor models, it limits experimental applications and precludes settings in which all cells in a tissue need to be manipulated uniformly to draw valid conclusions. An artificial bidirectional promoter was also applied by Wangensteen et al. to connect the expression of the Fah selection marker and the NRas^G12V^ oncogene, but the authors did not provide a detailed characterization of the amount of bidirectional transcripts relative to each other and to an endogenous control. A less effective liver repopulation as compared to our method can also be assumed potentially due to the application of less efficient SB transposase helper variants.

Here we used the hyperactive SB100 transposase helper for more effective chromosomal transgene delivery [11]. When designing our technology platform, we considered it important to allow tag-free expression of proteins of interest in such a way that their expression remains bound to the selection marker. The use of 2A-type peptides is not appropriate for this purpose, as they leave tags on both sides on the two transcriptionally linked proteins [17]. To overcome this challenge, we applied the HADHA/B endogenous bidirectional promoter capable of driving well-balanced bidirectional expression in the physiological range (Figs. 1 c and 2b). The use of HADHA/B gives the possibility for the marker-linked expression of an untagged native protein or a mutant protein isoform at the position of EGFP in transcript B (Fig. 1 a). Bidirectional promoters are more common than thought; a survey of the human genome indicated their widespread occurrence [44, 45]. The HADHA/B promoter is designed by nature to produce two subunits of a protein complex in stoichiometric proportions [18]. According to our own measurements, the ratio of the two driven transcripts is indeed closer to 1/1 than for any other bidirectional promoter reported so far (Figs. 1 c and 2b). In studies of either natural or synthetic bidirectional promoters, if such measurements have been performed at all, bidirectional transcript ratios worse than what we found for HADHA/B have been reported [46–50]. In a single case, we observed a significant shift from the typical 1/1 transcript A to B ratio to a ratio around 8/1 (Fig. 3d). Here the hRas^G12V^ CDS was likely to exert negative selection pressure on the B side of the promoter, while positive pressure was still present on its A side (Fah), and in turn, the connection between the two sides of the promoter was weakened. The 1/1 transcript ratio produced by the HADHA/B bidirectional promoter can be particularly important for protein production when attempting to produce protein complexes.

In addition, we aimed to optimize amiR-based gene silencing in vivo in mouse liver. Importantly, we wanted this feature to be included in the same construct from which the other gene of interest (GOI) is expressed and on which the Fah selection marker is present. Thus, all genetic features are jointly represented in all Fah-corrected liver cells. We have also aimed to position the amiR element in such a way that its maturation does not interfere with the expression of either the selection marker or GOI. In light of this, we decided to place our amiR elements in an intron modified for this purpose. It is well known that in vivo stable gene silencing is not a straightforward technology. The in vivo toxicity of shRNA expression was long ago reported [42]. Similarly, amiR-expressing germline transgenic mice frequently show toxicity and viability issues. Miura and co-workers reported that it was not possible to generate either transgenic mice with higher amiR-expressing constructs or to generate homozygous mice with lower amiR-expressing constructs [43]. The potential reason was the saturation of endogenous miR-processing pathways or other non-specific toxic side effects of the applied amiRs. All these toxic side effects were enhanced with expression level. Here we did not observe severe, non-specific toxicity of gene silencing. In livers expressing the amiR-EGFP element, transcript A levels were virtually identical to those measured in controls not carrying amiR elements. While RT-qPCR measurements revealed only a slight non-significant decrease in levels of transcripts A and B in groups of animals carrying one or two amiR-mP53 elements as compared to controls (Fig. 2b), this slight decrease could be explained by negative selection against strong *Tp53* silencing, which is supported by the decrease in transgene copy number detected in these organs (Fig. 2d). Consequently, the physiological level of transgene expression provided by the human *HADHA/B* promoter in vivo in the mouse liver is compatible with the application of amiR elements.

No sign of tumorigenesis was detected in mice treated with EGFP-expressing transposon constructs either with or without intronic amiR structures. Even in strongly *Tp53* silenced mice carrying double amiR-mP53 elements, no tumor induction was observed. It is worth comparing these data with the published phenotype of *Tp53* KO mice [51]. Heterozygous *Tp53* KO animals rarely developed only lymphoid and testis tumors by 9 months of age, whereas approximately 75% of homozygous KO mice developed various tumors by 6 months of age. The most frequently observed tumors were malignant lymphomas, but no liver tumors were observed at all. Our results are in line with this, as we do not expect the development of hepatocellular carcinoma (HCC) in our experimental system within the given monitoring period even after aggressive *Tp53* silencing. This also implies that SB transposon-mediated random transgene delivery has no or very low oncogenic side effects, meaning that it is not sufficiently tumorigenic by itself to ruin our test system even in the presence of *Tp53* silencing.

Previously, it was shown that upon NTBC withdrawal a small subpopulation of Fah-deficient hepatocytes may be able to avoid elimination during the selection process by activating the survival Akt pathway [52]. Presumably, this phenomenon is responsible for the emergence of tumors from non-corrected Fah-deficient cells reported during retroviral gene therapy treatment of *Fah* KO mice [53]. In our system, such tumors of tyrosinemic cell origin are well distinguishable from marker-positive tumors induced by our transgenes, as they are negative for the mCherry marker. Their development requires the long-term presence of a large number of residual, uncorrected Fah-deficient cells in the liver. The emergence of this can be attributed to two potential causes: the poor efficiency of gene delivery and the use of contraselective transgenes. With the use of highly contraselective transgenes, during multi-nodular repopulation, the growth of nodules expressing these transgenes more strongly is retarded, slowing down the overall repopulation of the liver. Using the hyperactive SB100 transposase helper and non-contraselective transgenes, we do not see these tumors. However, the use of contraselective transgenes may also induce their appearance in our system in the long term. Such mCherry marker-negative tumors emerging from non-corrected Fah-deficient cells were only detected in double amiR-mP53 carrying mice at late time points, around 7–10 months post-injection. Consistent with the fact that tyrosinemic cells are prone to cancerous transformation, our measurements revealed that they might exhibit increased levels of *Tp53* expression as well. *Tp53* expression was measured in control (no amiR, EGFP) animals at two time points, during multi-nodular repopulation, at 5 weeks after injection (Fig. 3c), and well after the completion of multi-nodular repopulation, at 5 months after injection (Fig. 2b). These RT-qPCR measurements revealed significantly higher *Tp53* expression levels in the first case (0.55-fold of Rpl27) at a considerable tyrosinemic (*Fah* KO) cell content than in the second case (0.34-fold of Rpl27), where tyrosinemic cells are already virtually absent.

Hepatocellular carcinoma (HCC) is one of the most lethal cancers worldwide; however, the genetic mechanisms underlying its pathogenesis are incompletely understood. We aimed to demonstrate the potential of our technology for cancer research by transforming mouse hepatocytes with the well-known *Tp53* tumor suppressor and *hRas* oncogene driver combination. Oncogenic hRas expression is able to trigger senescence in primary cells [54]. Therefore, their transformation by hR*as* requires either a cooperating oncogene or the inactivation of a tumor suppressor [54, 55]. In accordance, ectopic expression of hRas^G12V^ alone is insufficient to induce tumorigenesis. Thus, tumor development in our system alone demonstrates the combined manifestation of both cancer-driving genetic manipulations.

HCC characteristic of the induced tumors is well demonstrated by their Gpc3 and Afp immunoreactivity (Fig. 3a). It should also be noted that the presence of the Gpc3 marker, which can be used to distinguish early-stage HCC from dysplastic nodules [28], can already be detected in the majority of transformed hepatocyte colonies at the earlier examination time point, 5 weeks post-treatment (Fig. 3a).

The experimental potential created by our technology platform is not fully demonstrated by the *Tp53*-silenced and hRas^G12V^-overexpressing tumor model. For the application of this highly penetrant driver combination, wild-type mice are also suitable, as it is sufficient to produce a small number of cells here that survive p53 silencing and overexpress hRas^G12V^ to induce tumors. The high penetrance of oncogenic *Ras* mutations is a phenomenon parallel to the high incidence of *RAS* mutations in human cancer. Approximately 19% of patients with cancer harbor *RAS* mutations, equivalent to approximately 3.4 million new cases per year worldwide [56]. However, the current priority in cancer research is the functional validation of candidate driver genes with lower mutation frequencies in cancer genome databases. Such driver genes, when mutated, are likely to induce tumors at lower penetrance and may appear as germline or somatically mutated driver genes in hereditary as well as sporadic cancers [57]. The nearly 100 million testable hepatocytes available in our platform in a single experimental animal allow to functionally test even such driver genes with low-penetrance driver mutations.

## Conclusions

In this study, we developed a somatic gene delivery technology enabling long-lasting and high-level transgene expression in the entire hepatocyte population of mice. We also presented comparative studies to demonstrate that our approach is superior to conventional methods. Our technology allows the tag-free expression of proteins of interest and silencing of any arbitrary gene in the mouse genome using amiR elements. Achieving these has been aided by the use of the endogenous HADHA/B promoter capable of driving well-balanced bidirectional expression and by optimizing in vivo intronic amiR-based gene silencing. The HADHA/B promoter has not been part of the biotechnology toolkit until now. Here we provide a detailed characterization of its functionality in an in vivo setting. Eventually, we developed a versatile technology platform for in vivo somatic genome editing in the mouse liver that simultaneously meets multiple requirements. We expect it will contribute to gene function analysis in mice by generating new, more accurate genetic models.

## Methods

### Animal care and maintain

Mice were bred and maintained in the Central Animal House at the Biological Research Centre (Szeged, Hungary). The specific pathogen-free status was confirmed quarterly according to FELASA (Federation for Laboratory Animal Science Associations) recommendations [58]. Mice were housed under 12-h light-dark cycle at 22 °C with free access to water and regular rodent chow. All animal experiments were conducted according to the protocols approved by the Institutional Animal Care and Use Committee at the Biological Research Centre. The used *Fah* mutant line, C57BL/6N-*Fah*^*tm1(NCOM)Mfgc/Biat*^, is archived in the European Mouse Mutant Archive (EMMA) under EM:10787. *Fah*^−/−^ mice were treated with 8 mg/l Orfadin®(Nitisinone, NTBC) (Swedish Orphan Biovitrum) in drinking water. After hydrodynamic injection, NTBC was withdrawn. C57BL/6NTac wild-type mice were obtained from Taconic Biosciences.

### Plasmid construction

Empty pbiLiv-miR vector was synthesized and cloned in a pUC57 plasmid backbone by GeneScript. This encompasses the bidirectional promoter of the human hydroxyacyl-CoA dehydrogenase trifunctional multienzyme complex alpha (HADHA) and beta (HADHB) subunits. The HADHA side of the bidirectional promoter drives expression of the mCherry fluorescent marker gene, which is disrupted by the first intron of the human eukaryotic translation elongation factor 1 alpha 1 (*EEF1A1*) to ensure intronic expression of the designed amiR structures. Restriction endonuclease recognition sites were introduced into the EEF1A1 intron to clone amiR elements as follows: AgeI, XbaI, SacI, and SalI. The mCherry coding sequence (CDS) is linked to the mouse fumaryl-aceto-acetate dehydrogenase (*Fah*) CDS by a T2A peptide to provide bicistronic expression. The transcription unit ends with a bGH polyadenylation signal. The HADHB side of the bidirectional promoter is flanked by an MCS followed by a bGH polyadenylation signal. The whole arrangement is flanked by the T2 type SB transposon inverted terminal repeats [14].

To generate pbiLiv-miR-EGFP, the PCR amplified EGFP coding sequence was inserted into the BamHI/PacI sites of the MCS in pbiLiv-miR. pbiLiv-miR-EGFP-EGFP and pbiLiv-miR-mP53/1-EGFP were constructed by inserting the complete amiR-EGFP or amiR-mp53/1 element into the AgeI/XbaI site of pbiLiv-miR-EGFP. pbiLiv-miR-mP53/1,2-EGFP was constructed by inserting the complete amiR-mP53/2 element into the SacI/SalI sites of pbiLiv-miR-mP53/1-EGFP. Complete amiR-EGFP, amiR-mp53/1, amiR-mp53/2, and amiR-mp53/3 elements (Additional file 1: Fig. S1) flanked by AgeI, SacI, XbaI, and SalI sites were synthesized and cloned in a pUC57 plasmid backbone by GeneScript.

To generate pbiLiv-miR-Luc, the Luciferase encoding gene was amplified by PCR from plasmid pGL3-Basic (Promega) and inserted into the NheI/PacI sites of the MCS in pbiLiv-miR.

For constructing pbiLiv-miR-mP53/1-hRas^G12V^, hRas coding sequence was PCR amplified from mouse total liver RNA and cloned into the pBluescript SK plasmid. G12V mutation was introduced by the QuickChange Site Directed Mutagenesis Kit (Agilent Technologies). The mutated hRas coding sequence was then inserted into the BamHI/PacI sites of the MCS in pbiLiv-miR. Next, the amiR-mP53/1 element was inserted into the AgeI/XbaI sites of the hRas^G12V^-containing pbiLiv-miR.

pNeo-miR was constructed by inserting the first intron of the *EEF1A1* with the AgeI, XbaI, SacI, and SalI restriction endonuclease recognition sites into pT2-SVNeo (Addgene #26553). The pNeo-miR-mP53 plasmid series was constructed by inserting the amiR-mP53/1, amiR-mP53/2, and amiR-mP53/3 amiR structures into the AgeI/XbaI sites of pNeo-miR. pcGlobin2-SB100 was constructed as described [11].

### Hydrodynamic tail vein injection

Plasmids for hydrodynamic tail vein injection were prepared using the NucleoBond Xtra Maxi Plus EF Kit (Macherey-Nagel) according to the manufacturer’s instructions. Before injection, we diluted plasmid DNA in Ringer’s solution (0.9% NaCl, 0.03% KCl, 0.016% CaCl_2_) and a volume equivalent to 10% of mouse body weight was administered via the lateral tail vein in 5–8 s into 6–8-week-old mice [10, 59]. The amount of plasmid DNA was 50 μg for each of the constructs mixed with 4 μg of the transposase helper plasmid.

### RNA extraction and gene expression analysis

Total RNA from 50 mg liver tissue was isolated using TRI Reagent (MRC) following the manufacturer’s protocol. RNA was Dnase I treated with PerfeCTa DNase I (Quantabio) and reverse transcribed into cDNA using RevertAid First Strand cDNA Synthesis Kit (ThermoFisher Scientific). RT-qPCR was performed on a Rotor-Gene Q instrument (Qiagen) with PerfeCTa SYBR Green SuperMix (Quantabio) as follows: 95 °C for 7 min followed by 35 cycles of 20 s at 95 °C, 20 s at 60 °C, and 20 s at 72 °C. All reactions were carried out in triplicates in a final volume of 20 μl. The following primers were used:

- mP53-F: ACTTACCAGGGCAACTATGGCT; mP53-R: GCTGGCAGAATAGCTTATTGAGG;
- EGFP-F: GAGCAAAGACCCCAACGAGA; EGFP-R: CACCTCGAGCTACAGCTTCT;
- mFah-F: CTGGGTCAAGCTGCATGGAA; mFah-R: AGGAAGGTGCATTGTCGCAG;
- Rpl27-F: AAGCCGTCATCGTGAAGAACA; Rpl27-R: CTTGATCTTGGATCGCTTGGC [60].

PCR efficiencies were analyzed with Rotor-Gene Q software (Qiagen). Gene expression was analyzed by the normalization of expression to that of ribosomal protein L27 (*Rpl27*) using the ΔCT method [61].

### Genomic DNA isolation and transgene copy number assessment

Whole livers of treated animals were lysed in 150 ml lysis buffer (100 mM TRIS-HCl pH8, 5 mM EDTA pH8, 200 mM NaCl, 0.2% SDS) and incubated overnight at 50 °C in the presence of 300 μg/ml ProteinaseK (VWR Chemicals). DNA from 1 ml lysate was isolated by conventional phenol/chloroform extraction and ethanol precipitation.

The assessment of transgene copy number was done by qPCR. Primers for Fah were the same as for the analysis of mRNA amount. PerfeCTa SYBR Green SuperMix (Quantabio) was used to carry out qPCR reactions on a Rotor-Gene Q instrument (Qiagen). All reactions were carried out in triplicates using 30 ng gDNA. Cycling conditions were as follows: 95 °C for 7 min followed by 35 cycles of 20 s at 95 °C, 20 s at 64 °C, and 20 s at 72 °C. PCR efficiencies were analyzed with Rotor-Gene Q software (Qiagen). Relative changes in DNA levels were calculated using the ΔCT method. Results were normalized to measurements of the *Olfr16* gene. The following primers were used for this: Olfr16-F: GAGTTCGTCTTCCTGGGATTC; Olfr16-R: TAATGATGTTGCCAGCCAGA [62].

### Stereomicroscope imaging

Pictures of whole mouse livers were taken with an Olympus SZX12 fluorescence stereozoom microscope equipped with a 100-W mercury lamp and filter sets for selective excitation and emission of GFP and mCherry.

### In vivo bioluminescence imaging

In vivo bioluminescence imaging was performed using an IVIS Lumina III instrument (PerkinElmer). Following the intraperitoneal administration of 150 mg/kg luciferase substrate (XenoLight D-Luciferin-K+ salt, PerkinElmer), mice were anesthetized using isoflurane (Isoflurin, Vetpharma) and imaged at 10 min post-injection. Emitted photons were quantified with an exposure time of 1 to 10 s. Quantification of average radiance (photons per second per cm^2^ per steradian (sr) [p/s/cm^2^/sr]) within a circular region of interest was performed using the Living Image software (PerkinElmer).

### Immunohistochemistry

Mice were sacrificed at 5 weeks, 3 months, or 5 months post-injection. Livers were removed and fixed overnight in 4% formalin, then embedded in paraffin, and cut into 5-μm sections. Immunohistochemistry was performed using the EnVision FLEX Mini Kit (DAKO). Antigen retrieval was done in a PT Link machine (DAKO). The primary antibodies used for immunohistochemistry are rabbit polyclonal anti-FAH (ThermoFisher Scientific, PA5-42049, 1:100), incubated for 120 min, and rabbit polyclonal anti-alpha 1 fetoprotein (Abcam, ab46799, 1:500) and rabbit polyclonal anti-glypican 3 (Abcam, ab186872, 1:800), incubated overnight. Secondary antibody polyclonal goat anti-rabbit-HRP (DAKO, P0448) was incubated for 30 min. Visualization was done with EnVision FLEX DAB+ Chromogen System (DAKO, GV825). After hematoxylin counterstaining for 5 min, slides were mounted and scanned with a Pannoramic Digital Slide Scanner (3D Histech).

### Image analysis pipeline

3D Histech generated images were processed using BIAS software. A pipeline was created for the analysis consisting of four major steps: (1) pre-processing of the images, (2) segmentation and (3) feature extraction, and (4) cell classification using machine learning. In the pre-processing, non-uniform illumination was corrected using the CIDRE method [63]. The deep learning segmentation method was applied to detect and segment individual nuclei in images. With segmentation post-processing, two additional regions were defined for each nucleus: (1) a region representing the entire cell was defined by extending nuclei regions with a maximum 5 μm radius so that adjacent cells did not overlap and (2) cytoplasmic regions were defined by subtracting nuclei segmentation from the cell segmentation. Finally, morphological properties of these three different regions as well as intensity and texture features from all channels were extracted (in total 228 features) for cell classification. We employed supervised machine learning to predict four different cell types: FAH-positive cells, FAH-negative cells, immune cells, and other cells or segmentation artifacts that can be considered trash. These classes were manually selected based on their morphological characteristics. Cells with evenly distributed brown chromogen signal (anti-FAH staining) across the whole cells were labeled as FAH positive, while cells without chromogen staining were labeled as FAH negative. Cells with small and dark blue nuclei were considered as lymphocyte-like immune cells. Small segmented regions outside the tissue section were also classified as trash. For the training set, we annotated around 200 cells for each class from different tissue sections. Support Vector Machine (SVM) was trained with a radial basis function kernel commonly used for the multi-class cell phenotype classification. After training the SVM model, a 10-fold cross-validation was used to determine the expected accuracy of the model. We used this trained model to predict a class for all other cells in each liver section.

### Protein extraction and Western blotting

Fifty milligrams of liver tissue was dounce homogenized in 2 ml radioimmunoprecipitation assay (RIPA) buffer (10 mmol/l Tris-HCl, pH 8.0, 1 mmol/l EDTA, 0.5 mmol/l EGTA, 1% Triton X-100, 0.1% sodium de-oxycholate, 0.1% SDS, 140 mmol/l NaCl), supplemented with PMSF (Merck). Cleared samples were sonicated for 3×10 s. Protein concentrations were calculated using the Pierce^TM^ BCA Protein Assay kit (ThermoFisher Scientific). A total of 80 μg of protein was separated on 10% SDS-PAGE gel, transferred to a 0.2-μm nitrocellulose membrane (Amersham), and blocked with 5% non-fat dry milk in Tris-buffered saline-Tween 20 (TBS-T) for 1 h at room temperature. Blocked membranes were incubated with anti-GFP (Abcam, ab6556, 1:4000) and peroxidase-conjugated anti-GAPDH (ThermoFisher Scientific, MA5-15738-HRP,1:10000) antibodies. Anti-rabbit IgG conjugated to horseradish peroxidase (HRP) (Sigma Aldrich, A0545, 1:20000) was used as the secondary antibody where necessary. Immune complexes detected with enhanced chemiluminescence (ECL) Prime Western blotting Detection Reagent (Amersham).

### Cell culture and transfection

NIH/3T3 cells were purchased from ATCC and cultured in Dulbecco’s modified Eagle’s medium (DMEM) (Biosera) supplemented with 10% fetal bovine serum (FBS; Gibco) and 1% penicillin-streptomycin (P-S; HyClone) in the presence of 5% CO_2_ at 37 °C.

Cells were transfected with 500 ng of either pNeo-miR or pNeo-miR-mP53/1 or pNeo-miR-mP53/2 or pNeo-miR-mP53 in combination with 50 ng of the transposase helper plasmid, using FuGENE® HD transfection reagent (Promega) according to manufacturer’s instructions. Selection of stable transfected cells was performed using neomycin (G418; Biosera).

### Data visualization and statistics

GraphPad Prism software (version 8.4.3 for Windows, GraphPad Software) was used for data visualization. Statistics were calculated using Fisher’s exact and Pearson’s chi-squared test.

To identify levels of statistical significance (*P* value), one-way ANOVA tests were performed for the comparison of RT-qPCR measurements of different sample groups and for the comparison of two sample groups the Welch’s *t*-test was applied. The threshold for significance was *P*<0.05.

## Supplementary Information

**Additional file 1: Fig. S1.** The structure of the applied amiR elements. **Fig. S2.** Detection of the EGFP protein in mouse liver. **Fig. S3.** SB transposon-based cloning platform for the expression of amiR elements in cultured cells. **Fig. S4.** Stereomicroscopic and histological examination of liver samples 5 months after treatment with a construct mixture containing 1% transforming construct.

**Additional file 2.** Individual data values and statistics for Figures 1-3.

## Data Availability

All data generated or analyzed during this study are included in this published article and supplementary information files. Individual data values and statistics are provided in Additional file 2. Presented qPCR experiments comply with the MIQE Guidelines. Necessary details for evaluation are supplied in the “Methods” section of the manuscript. The exact sequences of artificial microRNA structures used are provided in Additional file 1: Fig. S1.

## Abbreviations

Afp: Alpha-fetoprotein
AIM2: Absent in melanoma 2
amiR: Artificial microRNA
CDS: Coding sequence
cGAS: Cyclic GMP-AMP synthase
EEF1A1: Eukaryotic translation elongation factor 1 alpha 1
EGFP: Enhanced green fluorescent protein
Fah: Fumarylacetoacetate hydrolase
GOI: Gene of interest
Gpc3: Glypican-3
HADHA/B: Hydroxyacyl-CoA dehydrogenase trifunctional multienzyme complex alpha and beta subunits
HCC: Hepatocellular carcinoma
IHC: Immunohistochemistry
ITR: Inverted terminal repeat
KO: Knock out
Luc: Firefly luciferase
miR: MicroRNA
NTBC: 2-(2-Nitro-4-trifluoromethylbenzoyl)-1,3-cyclohexanedione (Orfadin®, Nitisinon)
Rpl27: Ribosomal protein 27
RT-qPCR: Quantitative reverse transcription PCR
SB: Sleeping Beauty transposon
shRNA: Short hairpin RNA
TLR9: Toll-like receptor 9
WT: Wild type
