# Supplementary material for: A versatile transposon-based technology to generate loss- and gain-of-function phenotypes in the mouse liver
Source: BMC Biol. 2022 Apr 1;20:74. doi: 10.1186/s12915-022-01262-x (PMC8974095; doi:10.1186/s12915-022-01262-x)
Supplement: Supplementary file 1 — Additional file 1: Fig. S1. The structure of the applied amiR elements. Fig. S2. Detection of the EGFP protein in mouse liver. Fig. S3. SB transposon-based cloning platform for the expression of amiR elements in cultured cells. Fig. S4. Stereomicroscopic and histological examination of liver samples 5 months after treatment with a construct mixture containing 1% transforming construct. [file 12915_2022_1262_MOESM1_ESM.pdf]

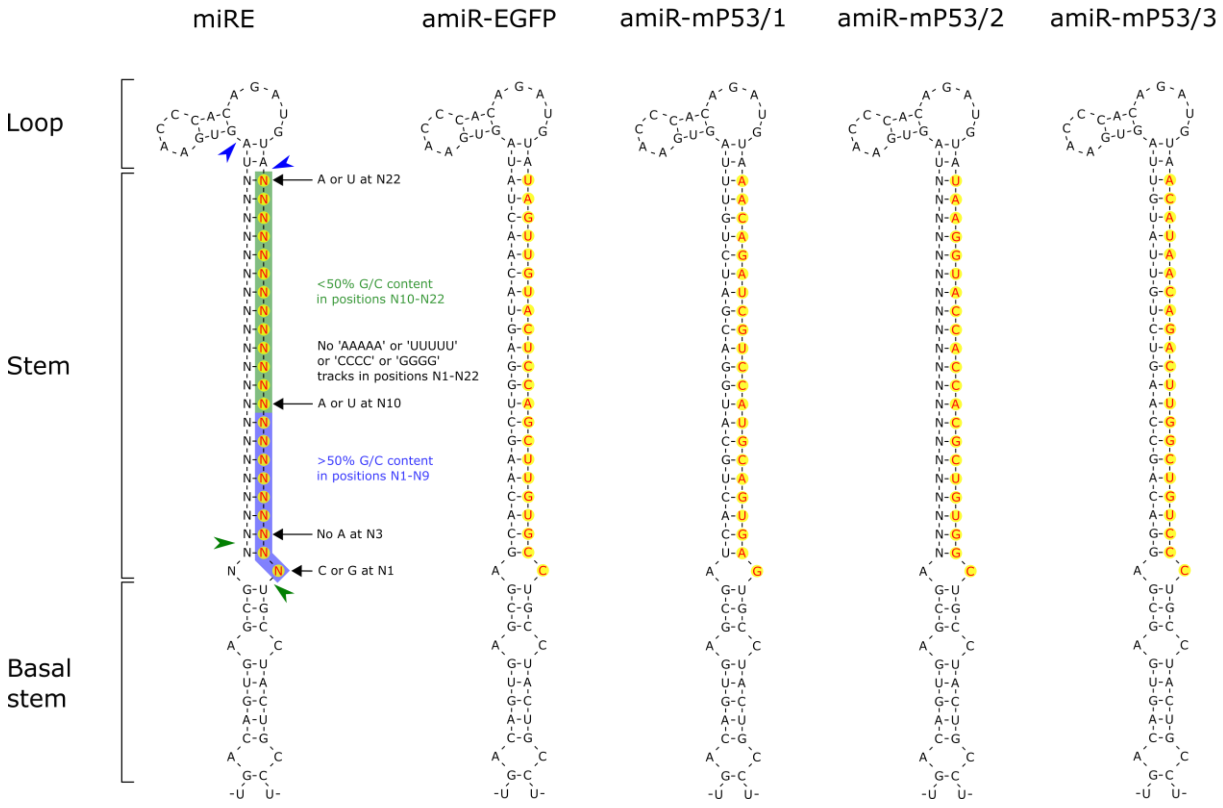

Figure S1 The structure of the applied amiR elements. Here we do not show the complete amiR structures, only the guide sequences and their sequence neighbourhoods. The selected guide sequences were incorporated into 'miR-E' an optimized human miR-30a-based miR backbone [20]. For guide sequence selection we followed the guide design rules of Dow et al. [21]. The rules we followed are summarized on the left side of the figure in a 'miR-E' context. Guide sequences are written in red on a yellow background. Green arrows indicate the cleavage site of the Drosha protein and blue arrows indicate the cleavage site of the Dicer protein.

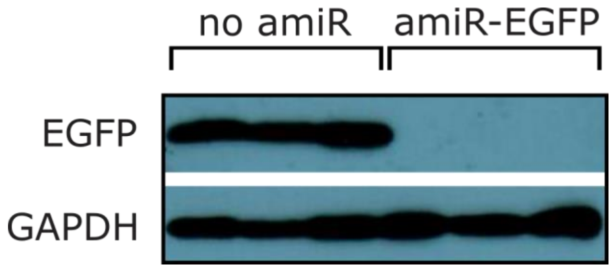

Figure S2 Detection of the EGFP protein in amiR-free control (no amiR, EGFP) or amiR-EGFP-expressing (amiR-EGFP, EGFP) transposon vector treated *Fah*$^{-/-}$ mice. Liver extracts prepared at 5 months post-treatment were tested by Western blot assay for the presence of EGFP protein. GAPDH served as a loading control.

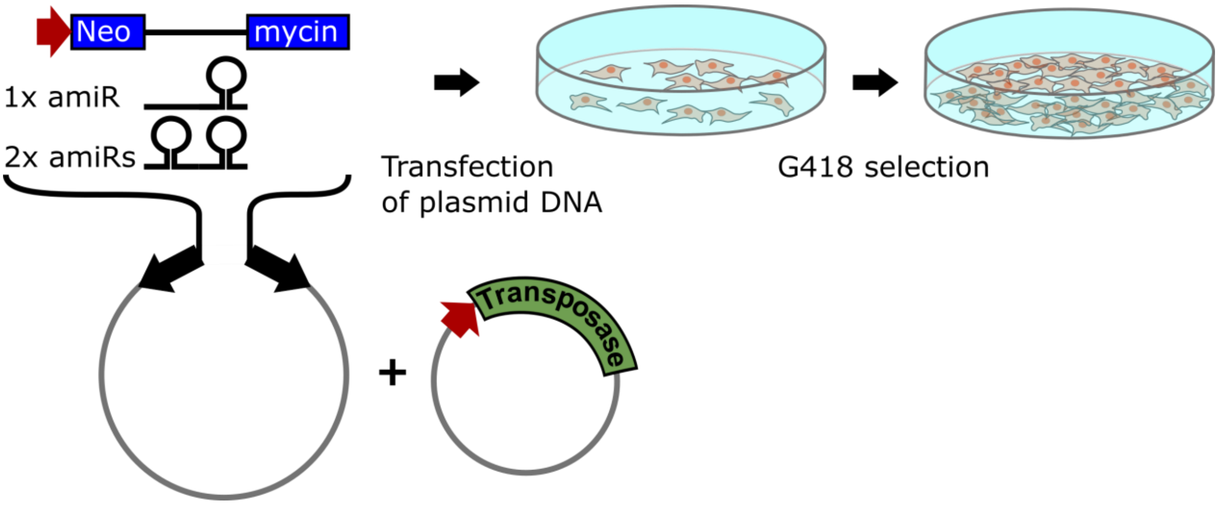

Figure S3 Schematic representation of the SB transposon-based cloning platform for the expression of amiR elements in cultured cells (pNeo-miR) and outline of the cell culture experiments.

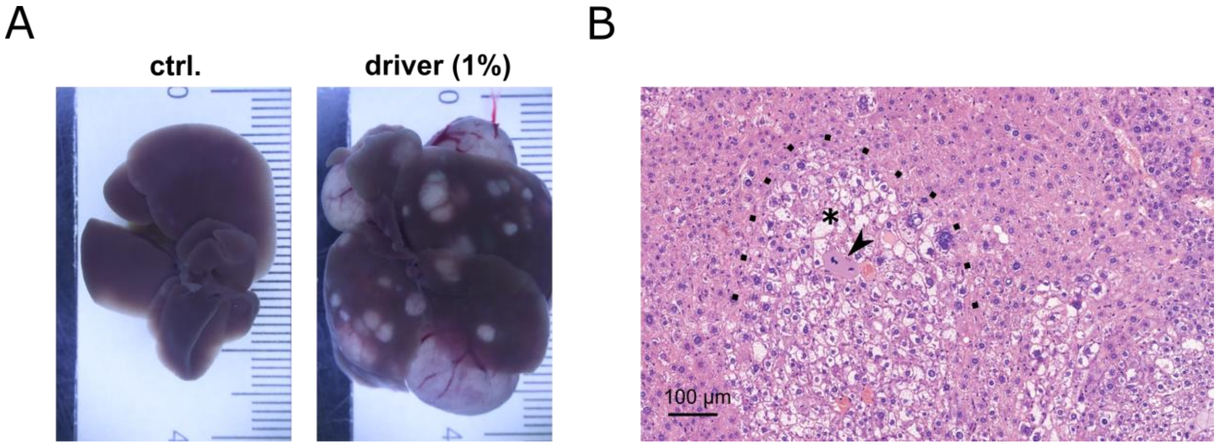

Figure S4 Investigation of HCC developed 5 months after treatment in *Fah*$^{-/-}$ animals treated with a construct mixture containing 1% transforming construct. **a** Representative stereomicroscopic images of the liver of *Fah*$^{-/-}$ animals treated either with the control construct alone (no amiR, EGFP) or with a 1 in 100 mixture of the driver construct (amiR-mp53/1, hRas^{G12V}) and a Fah selection marker expressing construct. Mice were sacrificed at 5 months after NTBC withdrawal. Stereomicroscopic images showed that the livers of animals treated with low-dose of driver construct had high tumor burden. **b** Hematoxylin/Eosin staining of a representative area of HCC developed 5 months after treatment in *Fah*$^{-/-}$ animals treated with a 1 in 100 mixture of the driver construct (amiR-mp53/1, hRas^{G12V}) and a Fah selection marker expressing construct. Intracellular lipid accumulation frequently observed in tumor tissue is a typical aspect of HCC (asterisk). A region of neoplastic cells invading surrounding liver tissue (dotted line) and an atypical mitosis (black arrowhead) are also marked.
